# Supplementary material for: Long-Term Phenotypic and Proteomic Changes Following Vitrified Embryo Transfer in the Rabbit Model
Source: Animals (Basel). 2020 Jun 17;10(6):1043. doi: 10.3390/ani10061043 (PMC7341293; doi:10.3390/ani10061043)

S1 Figure. Pie charts showing the distribution of the differentially expressed proteins between animals born from vitrified-transferred embryos and those conceived naturally based on their molecular function, biological process and cellular component.

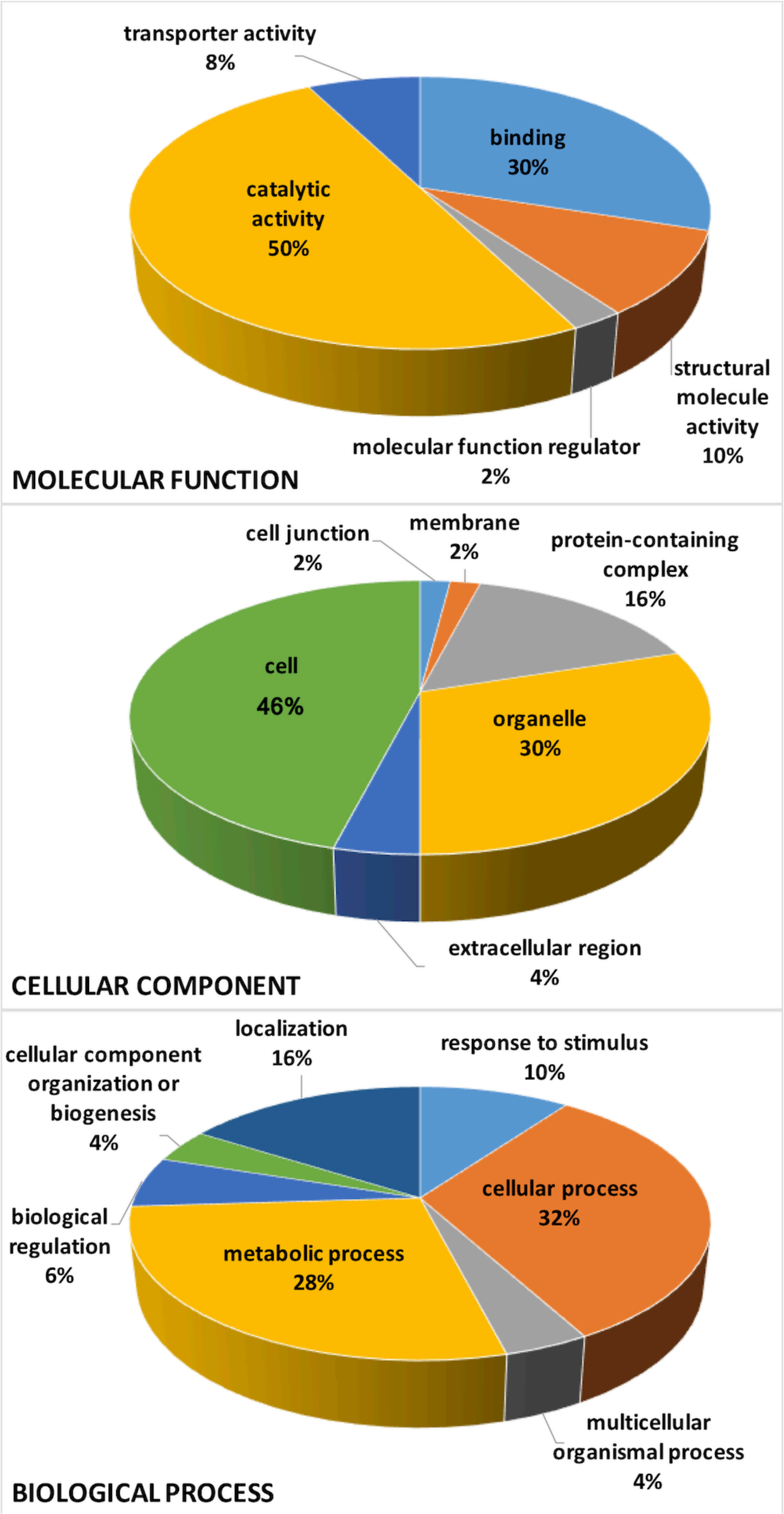

Supplement: Supplementary file 1 [file animals-10-01043-s001.zip › S1 figure.pdf]
