# Supplementary material for: Long-Term Phenotypic and Proteomic Changes Following Vitrified Embryo Transfer in the Rabbit Model
Source: Animals (Basel). 2020 Jun 17;10(6):1043. doi: 10.3390/ani10061043 (PMC7341293; doi:10.3390/ani10061043)
Supplement: Supplementary file 1 [file animals-10-01043-s001.zip › S1_Table.pdf]

S1 Table. Differentially expressed proteins in liver tissue between animals born from vitrified-transferred embryos and those conceived naturally.

Uniprot accession	Gene name	Ratio*
G1TQG1	NADH:ubiquinone oxidoreductase subunit B9(NDUFB9)	-2,131
G1TM55	ribosomal protein S6(RPS6)	-1,849
G1TH09	60S ribosomal protein L7a(LOC100341006)	-1,755
G1TT27	ribosomal protein L8(RPL8)	-1,751
G1SVW5	ribosomal protein L4(RPL4)	-1,637
G1TFX2	alpha-1-antitrypsin(LOC100328621)	-1,583
G1T4E7	signal peptidase complex subunit 2(SPCS2)	-1,470
G1TUC2	CCHC-type zinc finger nucleic acid binding protein(CNBP)	-1,402
G1U6B4	ATP synthase subunit e, mitochondrial(LOC108178113)	-1,390
G1SN00	kininogen 1(KNG1)	-1,349
G1T9I4	sorcin(SRI)	-1,237
G1SEH7	NADH:ubiquinone oxidoreductase subunit B8(NDUFB8)	-1,212
G1TKV4	histone H3(LOC103350067)	-1,157
G1SLD5	hypoxia up-regulated 1(HYOU1)	-1,092
G1SCT1	prolyl endopeptidase(PREP)	-1,059
U3KMP8	myosin IB(MYO1B)	-0,996
G1TJW1	40S ribosomal protein S8(LOC100352057)	-0,987
G1U9R4	apolipoprotein B(APOB)	-0,968
G1SML9	DnaJ heat shock protein family (Hsp40) member A1(DNAJA1)	-0,938
G1U416	asialoglycoprotein receptor 1(ASGR1)	-0,931
O62648	sulfotransferase family 2A member 1(SULT2A1)	-0,884
G1TZQ6	NADH:ubiquinone oxidoreductase subunit A10(NDUFA10)	-0,868
G1TCW2	osteoclast stimulating factor 1(OSTF1)	-0,846
G1TM29	aldehyde dehydrogenase 16 family member A1(ALDH16A1)	-0,796
G1TSY8	alpha-1-microglobulin/bikunin precursor(AMBP)	-0,780
G1TPL7	dipeptidyl peptidase 3(DPP3)	-0,691
G1SE10	2-hydroxyacyl-CoA lyase 1(HACL1)	-0,686
G1TYY5	LIM and SH3 protein 1(LASP1)	-0,665
G1T7G4	growth arrest specific 2(GAS2)	-0,625
G1SYV9	talin 1(TLN1)	-0,613
G1T6D1	ribosomal protein L23(RPL23)	-0,597
G1U6X6	heterogeneous nuclear ribonucleoprotein H2(LOC100339065)	-0,548
G1SYT7	peptidase, mitochondrial processing beta subunit(PMPCB)	-0,533
G1SHF3	nitrilase 1(NIT1)	-0,517
G1TCE9	hydroxysteroid dehydrogenase like 2(HSDL2)	-0,504
G1T7T6	USO1 vesicle transport factor(USO1)	-0,498

G1SZT8	SEC13 homolog, nuclear pore and COPII coat complex component(SEC13)	-0,492
B2ZDY6	t-complex 1(TCP1)	-0,457
O77768	heterogeneous nuclear ribonucleoprotein C (C1/C2)(HNRNPC)	-0,439
G1SNB1	carboxylesterase 3(CES3)	-0,412
G1U7C5	ribosome binding protein 1(RRBP1)	-0,404
G1T3D7	N-acetylneuraminate synthase(NANS)	-0,396
G1SZ47	ribosomal protein S23(RPS23)	-0,378
A0A0G2JH20	heat shock protein 90 alpha family class A member 1(HSP90AA1)	-0,336
G1SCN8	chaperonin containing TCP1 subunit 3(CCT3)	-0,297
G1TBS1	Parkinsonism associated deglycase(PARK7)	-0,292
G1U7L4	heat shock protein family A (Hsp70) member 5(HSPA5)	-0,292
G1U032	StAR related lipid transfer domain containing 10(STARD10)	-0,175
G1SKT4	ATP synthase, H+ transporting, mitochondrial F1 complex, alpha subunit 1, cardiac muscle(ATP5A1)	0,193
G1TS42	amylo-alpha-1, 6-glucosidase, 4-alpha-glucanotransferase(AGL)	0,557
G1SP40	amidohydrolase domain containing 1(AMDHD1)	0,687
G1SR29	ATPase H+ transporting V1 subunit A(ATP6V1A)	0,689
G1T3R4	alcohol dehydrogenase class-2 isozyme 1(ADH2-1)	0,705
G1T295	epoxide hydrolase 1(EPHX1)	0,766
G1T8P1	aldehyde dehydrogenase 1 family member L1(ALDH1L1)	0,832
Q75NJ2	aldehyde dehydrogenase 1 family member A1(ALDH1A1)	0,951
G1U0Z4	agmatinase(AGMAT)	0,959
G1TY06	glutathione S-transferase mu 2 (muscle)(GSTM2)	1,249
B7NZF9	nucleophosmin (nucleolar phosphoprotein B23, numatrin)(NPM1)	1,719
G1SZH0	retinol binding protein 4(RBP4)	1,872
G1SMM7	small nuclear ribonucleoprotein D3 polypeptide(SNRPD3)	2,211
G1U1M3	aminoacyl tRNA synthetase complex interacting multifunctional protein 1(AIMP1)	2,351
P04068	epoxide hydrolase 1(EPHX1)	2,648
G1TCQ2	fatty acid binding protein 1(FABP1)	2,754
G1TNI4	glutathione S-transferase Yb-3(LOC100357148)	2,921
G1TMP1	keratin, type I cytoskeletal 18(LOC100008885)	3,948

*Ratio represents the fold change (vitrified-transferred/naturally conceived).
