# Supplementary material for: Long-Term Phenotypic and Proteomic Changes Following Vitrified Embryo Transfer in the Rabbit Model
Source: Animals (Basel). 2020 Jun 17;10(6):1043. doi: 10.3390/ani10061043 (PMC7341293; doi:10.3390/ani10061043)
Supplement: Supplementary file 1 [file animals-10-01043-s001.zip › S2_Table.pdf]

S2 Table. Functional analysis of differential expressed proteins in liver tissue between animals born from vitrified-transferred embryos and those conceived naturally.

Category*	Term	Count	p-value
BP	toxin transport	4	0,000
BP	mitochondrial electron transport, NADH to ubiquinone	3	0,001
BP	translation	6	0,002
BP	protein folding	4	0,006
BP	10-formyltetrahydrofolate catabolic process	2	0,010
BP	positive regulation of protein localization to Cajal body	2	0,041
BP	retinol metabolic process	2	0,056
BP	positive regulation of telomerase RNA localization to Cajal body	2	0,066
BP	protein stabilization	3	0,071
BP	ATP synthesis coupled proton transport	2	0,080
BP	one-carbon metabolic process	2	0,090
BP	biosynthetic process	2	0,094
BP	binding of sperm to zona pellucida	2	0,099
CC	extracellular exosome	34	0,000
CC	myelin sheath	7	0,000
CC	mitochondrial respiratory chain complex I	4	0,000
CC	cell body	4	0,001
CC	cytosol	10	0,003
CC	membrane	10	0,006
CC	focal adhesion	6	0,010
CC	endoplasmic reticulum chaperone complex	2	0,029
CC	zona pellucida receptor complex	2	0,038
CC	chaperonin-containing T-complex	2	0,043
CC	cytoplasm	15	0,076
MF	aldehyde dehydrogenase (NAD) activity	3	0,000
MF	structural constituent of ribosome	6	0,003
MF	poly(A) RNA binding	11	0,008
MF	hydroxymethyl-, formyl- and related transferase activity	2	0,011
MF	formyltetrahydrofolate dehydrogenase activity	2	0,011
MF	proton-transporting ATPase activity, rotational mechanism	2	0,055
MF	ATPase activity	3	0,060
MF	peptidase activity	2	0,071

MF	glutathione transferase activity	2	0,091
MF	NADH dehydrogenase (ubiquinone) activity	2	0,097
KEGG	Ribosome	7	0,001
KEGG	Metabolism of xenobiotics by cytochrome P450	5	0,001
KEGG	Chemical carcinogenesis	5	0,003
KEGG	Oxidative phosphorylation	5	0,007
KEGG	Parkinson's disease	5	0,010
KEGG	Protein processing in endoplasmic reticulum	5	0,015
KEGG	Drug metabolism - cytochrome P450	3	0,072
KEGG	Alzheimer's disease	4	0,074
KEGG	Huntington's disease	4	0,099

*Functional analysis was referred to the GO term annotation according to the biological process (BP), cellular component (CC) and molecular function (MF) classification, and the KEGG pathways in which they are involved.
